# Evaluation of Apple Pomace as a Circular Feed Ingredient for *Tenebrio molitor* Larvae

**DOI:** 10.3390/insects16121208

**Published:** 2025-11-27

**Authors:** Michelle L. DuVall, Ida Holásková, Yong-Lak Park, Cangliang Shen, Jacek Jaczynski, Kristen Matak

**Affiliations:** 1 School of Agriculture and Food Systems, West Virginia University, P.O. Box 6108, Morgantown, WV 26506, USA; mlduvall@cox.net (M.L.D.); yong-lak.park@mail.wvu.edu (Y.-L.P.); cashen@mail.wvu.edu (C.S.); 2 Office of Statistics and Data Analytics, West Virginia Agricultural and Forestry Experiment Station, Davis College of Agriculture and Natural Resources, West Virginia University, Morgantown, WV 26506, USA; ida.holaskova@mail.wvu.edu

**Keywords:** yellow mealworm, edible insect(s), growth rate, proximate composition

## Abstract

Worldwide production of apple juice results in several million tons of apple pomace (AP) produced as a byproduct. The objective of this study was to determine whether the incorporation of AP into the diet of mealworm larvae (*Tenebrio molitor*) would influence growth performance, survival, and nutritional composition relative to standard feed. After 4 weeks of ad libitum access to commercial mealworm feed and non-nutritive water-storing beads (WB) or AP, no significant differences in weight gain or overall survival were measured. In both cohorts, higher pupation rates were associated with greater mortality among the remaining larvae; however, the weekly probability of survival was better in the AP cohort, potentially due to antioxidants present in apples. When measured on a dry matter basis, protein (34.4 vs. 33.8%), fat (51.9 vs. 52.2%), and ash (0.20 vs. 0.16%) levels did not differ significantly between the WB and AP cohorts, respectively. However, the WB cohort had more moisture (66.9 vs. 63.9%) likely because water beads provide a more direct source of hydration. These findings show that apple pomace, traditionally viewed as a waste product, shows promise in industrial applications.

## Simple Summary

This study explored how adding apple pomace to the diet of mealworm larvae affects their growth, survival, and nutritional composition. Mealworms are a sustainable, high-protein food source that require fewer resources than traditional livestock. The results showed that larvae fed apple pomace grew and developed just as well as those on a standard diet, with slightly higher survival rates. These findings suggest that apple pomace can be safely and effectively used as a low-cost ingredient in insect farming. By repurposing food industry waste into nutritious insect feed, this approach supports sustainable food production and helps reduce agricultural waste.

## 1. Introduction

Insects, particularly mealworm larvae, are abundant, require less land and water than traditional livestock, and have a significantly smaller carbon footprint [1]. Mealworms offer high-quality protein with all the essential amino acids, along with essential fatty acids such as linolenic acid, and other key nutrients [2]. Furthermore, mealworm larvae can efficiently convert agricultural residues and food industry by-products, including materials originating from food waste, into valuable biomass [2,3,4]. For instance, Bordiean et al. (2022) demonstrated that larvae reared on diets supplemented with wheat bran, rye bran, rapeseed cake, flax and milk thistle cake achieved an efficiency of conversion of ingested feed (ECI) that exceeded 45% [2]. Nutritional analysis of the dried larvae showed that combining wheat bran with these byproducts significantly increased the crude protein content. In a separate study using mealworms, Bordiean et al. (2022) reported that supplementing chicken feed with rapeseed meal improved the ECI compared to chicken feed alone [3]. Similarly, supplementing mealworm larvae diets with olive or carrot pomace has been shown to enhance growth performance, reduce mortality, and accelerate development [5,6]. For example, Ruschioni et al. (2020) found that replacing wheat flour with wheat middlings, or middlings supplemented with olive pomace (3:1), improved larval development time, pupal weight, and protein content [5]. Likewise, Rovai et al. (2021) reported that incorporating carrot pomace into a wheat bran-based diet reduced mortality and shortened pupation time, though weight gain only improved when carrot pomace comprised 20% or less of the feed [6]. Collectively, these studies highlight the potential of mealworms to upcycle low-cost agricultural by-products into valuable, protein-rich sources [4].

One such underutilized by-product is apple pomace, which is produced in vast quantities during apple juice production. In 2023, global apple production surpassed 83 million metric tons [7], generating over 4 million tons of apple pomace annually from the apple juice industry [8]. Composed primarily of peel and pulp, apple pomace is an excellent source of antioxidants and dietary fibers and can improve the nutritional profile of fortified foods [9]. Despite these nutritional benefits, much of the apple pomace generated is discarded as waste, creating an opportunity for valorization as a sustainable insect feed ingredient [3].

While the potential of agricultural by-products as mealworm feed has been demonstrated for material such as rapeseed, olive, and carrot pomace, research evaluating apple pomace as a feed supplement for mealworms remains limited, despite apples being among the most widely consumed fruits globally [10]. Therefore, the objective of this study was to evaluate the impact of incorporating apple pomace into mealworm diets on growth performance, survival, weight gain, and proximate composition, relative to larvae fed a standard wheat-based diet. By addressing this gap, our findings provide new evidence on the feasibility of using high-moisture fruit by-products in insect rearing systems, with broader implications for food waste reduction and the sustainability of alternative protein production.

## 2. Materials and Methods

### 2.1. Mealworm Maintenance

Medium-sized mealworm larvae were obtained from New York Worms (Long Island, NY, USA) and delivered to the Food Science Laboratory at West Virginia University (Morgantown, WV, USA). Two independent deliveries, spaced a few weeks apart, were used as independent experimental replicates. Each delivery served as a randomized replicate and was included as a blocking factor in subsequent data evaluation. When the larvae were received, they were transferred with the initial packaging material (crumpled newspaper) into a large holding container and unrestricted access to dry food (Wormy Worms Premium Mealworm Superworm Bedding Chow, Humble, TX, USA). The container was sealed with clear plastic film that was perforated to allow air flow, and the larvae were kept undisturbed in the dark at an ambient temperature for a period of 7 days.

To maintain control over freshness and uniformity, apple pomace was prepared on-site. Commercially grown organic Gala apples from the state of Washington were bought at a local grocery store (Wal-Mart, Morgantown, WV, USA). Prior to processing, the apples were rinsed, and the core was removed. A hand-operated fruit grinder (Fruit Apple Crusher, EJWOX, Wilmington, NC, USA) with rotating steel blades was used to slice and crush the prepared apples. Crushed apples were placed in cheesecloth and hand-pressed to remove excess juice and moisture. The resulting apple pomace contained approximately 86% moisture and was portioned into 25-g aliquots, sealed in Ziplock bags (SC Johnson, Racine, WI, USA), and stored at 0 °C. All pomace originated from this single, freshly prepared batch and was thawed in portions as needed.

### 2.2. Insect Growing Parameters

Following the acclimation period, the original packing material was discarded, and larvae were randomly and evenly distributed into 12 BPA-free, food-grade polyethylene freezer boxes (9 × 9 × 5 cm; Arrow Home Products, Elk Grove Village, IL, USA). Each container represented one experimental unit and served as a replicate for statistical analysis. Across both experimental shipments (n = 2), approximately 4774 larvae were distributed evenly, with 2386 allocated to the apple pomace (AP) treatment and 2388 to the water bead (WB) control. Each shipment of larvae was divided between 12 containers (6 per treatment) that held an average of 166 larvae (range: 107–286). This corresponds to an approximate density of 0.41 larvae/cm^3^ (410 larvae/L), which falls within the range reported to support normal growth and development in *Tenebrio molitor* without inducing crowding stress [11,12].

Uncovered boxes were housed in three separate incubators, four per unit (Thermo Scientific 18 L Low Temp Incubator (Thermo Fisher Scientific, Waltham, MA, USA) & Benchmark Scientific H2200-H My Temp Mini Incubator (Benchmark Scientific, Inc., Sayreville, NJ, USA)). To reduce variability across rearing environments, box positions were shifted among incubators and cycled between internal shelves twice per week. Incubator conditions were maintained at a target air temperature of approximately 27.8 °C, with fluctuations between 26.6 and 29.5 °C. Relative humidity averaged around 47%, ranging from 29.3 to 71.0%. Environmental conditions inside each incubator were tracked using a digital temperature and humidity hygrometer (Veanic Mini, model #8541833457, Shenzhen Aoyu Technology Co., Ltd., Shenzhen, China). The room housing the incubators primarily remained dark, with light exposure limited to less than one hour per day during routine feeding and maintenance.

### 2.3. Nutritional Interventions

All larvae were provided unlimited access to a commercially available dry feed for mealworms (Wormy Worms Premium Mealworm Superworm Bedding Chow, Humble, TX, USA). The feed consisted of wheat bran, corn flour, soybean flour, Brewer’s yeast, bone meal, and a blend of vitamins. To maintain cleanliness and reduce waste (frass) buildup, the dry feed and bedding material were replaced weekly.

Two cohorts were formed by randomly allocating the larvae. The control cohort (WB) received 15 g/week of water-retaining polymer crystals that offered hydration without nutritional value. The experimental cohort (AP) was provided with unlimited apple pomace as a moisture and nutrient supplement. To minimize drying and spoilage, the apple pomace was refreshed twice weekly.

### 2.4. Growth Performance

Larvae were inspected daily, and mortality was recorded as the number of visibly dead individuals removed from each container. Pupation was identified by the presence of immobile pupae with a white exoskeleton. The cumulative numbers of dead and pupated larvae were recorded weekly for each container. Because each container was the experimental unit, these data were averaged within containers for analysis. Additionally, ten larvae were randomly selected from each box and weighed weekly to monitor growth; these individual larvae served as subsamples to estimate the container mean weight.

Following the full experimental timeline, which included a 1-week acclimation phase followed by 4 weeks of dietary treatment, both the feed and hydration sources (water beads or apple pomace) were withdrawn for a 24-h gut-clearing period. After this fasting phase, larvae were euthanized by freezing. A subset of the frozen larvae was retained to determine moisture concentration, while the remainder underwent freeze-drying (FreeZone 8 L Tray Freeze Dryer, Labconco, Kansas City, MO, USA) and stored at −20 °C until further testing.

### 2.5. Proximate Composition

An analysis of proximate composition (crude protein, fat, moisture, and ash) was conducted on the commercial mealworm feed, apple pomace, and both frozen and freeze-dried mealworm larvae post-treatment. Crude protein levels were measured via Kjeldahl titration, applying a nitrogen-to-protein conversion factor of 5.6 [13]. Crude lipid was measured by extracting fat using the Soxhlet technique with petroleum ether (10 mL/min, 24 h) and then dried at 110 °C. Moisture was determined by weighing samples before and after drying for 24 h in an oven at 110 °C. Ash content of the samples were assessed by incineration at 550 °C for 24 h in a muffle furnace [14].

### 2.6. Experimental Design

Statistical evaluation was conducted using JMP Pro 16.0.0 and SAS 9.4 (SAS Institute Inc., Cary, NC, USA). Survival was assessed using three methods: repeated measures ANOVA (treating each box as the experimental unit; n = 6 per treatment), and two categorical approaches—the Life Table (actuarial) and Kaplan–Meier methods [15]. The mixed-model ANOVA included week as a repeated factor (autoregressive covariance structure), treatment cohort (WB, AP) as a fixed effect, and covariates such as pupation rate, humidity, and temperature. Interactions between treatment × week and pupation rate × week were also evaluated.

The categorical survival analyses generated weekly survival probabilities per larva, treating pupated individuals as censored. Larvae were removed upon pupation, and survival counts adjusted accordingly. Both methods assumed pupation occurred uniformly and independently of treatment. A Log-Rank test compared survival distributions between treatment cohort, with significant *p*-values indicating treatment effects. The relationship between pupation and mortality was further examined using Spearman’s (ρ) rank correlation.

For growth (weight), normality and variance homogeneity were tested using the Shapiro–Wilk and Levene’s tests, respectively. A repeated measures ANOVA was conducted with week as the repeated factor and treatment and treatment × week interaction as fixed effects. ANOVA was used to conduct an additional analysis of the final larval weight at week 4, accounting for study replication as a random factor. Because growth was analyzed using an ANOVA model, results are reported as least squares means (LS Means) ± standard error (SE), which reflects the precision of the model-adjusted means and facilitates direct comparison between treatments. One-way ANOVA was used to analyze proximate composition data (moisture, protein, fat and ash) to assess the effect of diet treatment (AP vs. WB), with Tukey–Kramer post hoc testing. For these analyses, values are presented as arithmetic means ± standard deviation (SD) to represent the variability within each treatment group. Statistical significance was predetermined at α = 0.05 for all analyses.

## 3. Results

### 3.1. Larval Growth, Weight Dynamics, and Survival Outcomes

The growth of the larvae was assessed by tracking the mean weight (g) of mealworms in each treatment cohort (water bead; WB and apple pomace; AP). Between the two cohorts, differences in weight gain were not significantly different over the 4-week period (*p* > 0.05) (Table 1). WB larvae showed an average weight increase of 84.99% ± 0.21, while AP larvae exhibited a similar gain of 81.86% ± 0.24.

**Figure 1:**
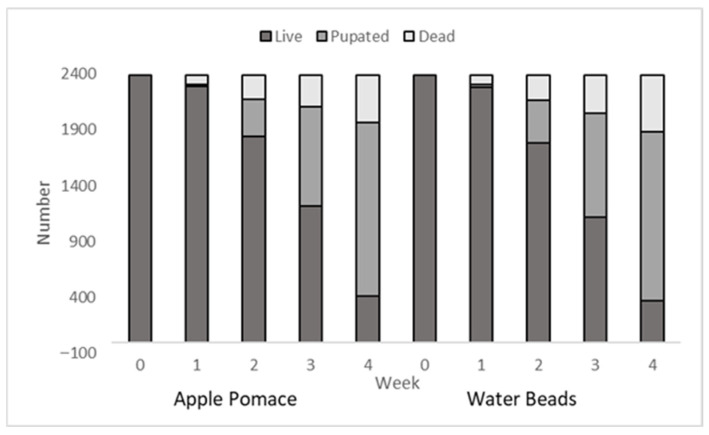
Number of live, pupated and dead mealworms fed a wheat-based worm diet supplemented with apple pomace (**left**, n = 2386) in place of standard water beads (**right**, n = 2388) over a 4-week period.

At the study’s conclusion, pupation rates increased to 24% and 28% for the WB and AP cohorts, respectively (Figure 1 and Table 2). Using repeated measures ANOVA while controlling for pupation rate, humidity, and temperature, the survival rates between the AP and WB groups did not significantly differ (*p* > 0.05). However, a significant association emerged between pupation frequency and survival in both cohorts (Spearman’s ρ < 0.05), with higher pupation rates corresponding to increased mortality.

The Life Table analysis showed survival rates of 66.71% for larvae provided with water beads and 73.28% for those fed apple pomace over the four-week treatment period (Table 2). There was a significant difference between cohorts (χ^2^ = 12.7954, DF = 1, *p* < 0.001), according to the Log-Rank Test for Equality of Survival Functions, indicating that the larvae in the apple pomace cohort had better survival than those in the water bead cohort.

### 3.2. Proximate Composition

The crude protein, crude lipid, moisture, and ash of the worm feed, apple pomace, and each cohort of mealworm larvae are reported in Table 3. The worm feed had a protein concentration of less than 9%, whereas the apple pomace contained approximately 1.2% protein. Mealworm larvae had an average crude protein concentration of 38.36 ± 0.06 g/100 g, with no significant differences between AP and WB treatments (*p* > 0.05; Table 3).

The average fat concentration across cohorts was 52.11 ± 0.12 g/100 g, and ash content was 0.18 ± 0.00 g/100 g. Moisture content differed significantly (*p* < 0.05), with WB larvae having higher levels (66.89 ± 0.01 g/100 g) than the AP group (63.89 ± 0.01 g/100 g). 

## 4. Discussion

As the global population continues to grow, identifying sustainable protein sources becomes increasingly important, particularly in comparison to traditional protein-rich foods such as lean meats, eggs, and legumes. Edible insects, including *T. molitor* larvae, represent a promising alternative, offering crude protein levels ranging from 47–60%, which often exceed those of common plant proteins such as soy, lentils, and grains [16].

These results suggest that incorporating apple pomace into the larval diet does not negatively affect growth rate or nutrient composition. The comparable weight gain between treatments aligns with findings by Benítez-González et al. (2024), who also observed no significant differences in growth rates when mealworms were fed various agro-industrial residues [17]. As in that study, the relatively short monitoring period and ongoing pupation may have limited differences in growth response. Future studies should therefore begin feeding immediately post-hatching and continue through pupation to capture longer-term effects.

Interestingly, although growth did not differ, survival was consistently higher among larvae receiving apple pomace, which may be partly explained by the antioxidant properties of this by-product. Apple pomace is rich in polyphenols, flavonoids, and other antioxidant compounds capable of scavenging free radicals and mitigating oxidative stress in biological systems [8,9]. During pupation, insects undergo rapid physiological and metabolic transformations that increase energy demand and elevate oxidative stress, often resulting in higher mortality rates [18,19]. In this context, the inclusion of antioxidant-rich apple pomace may have provided a protective effect by stabilizing redox balance and reducing pupation-related stress. Similar benefits have been reported for other plant-derived by-products rich in bioactive compounds, such as olive and carrot pomace, which enhanced larval performance and resilience in *T. molitor* [5,17]. Collectively, these findings suggest that bioactive compounds in fruit-based residues may give physiological advantages that support growth and survival during energetically demanding developmental transitions. While the antioxidant explanation remains hypothetical, it aligns with prior evidence that oxidative stress increases markedly during insect metamorphosis and that dietary antioxidants can modulate redox balance and gut microbial composition, improving resilience in *T. molitor* and other holometabolous insects [5,18]. Future work should include biochemical assays and microbiota analyses to validate these potential mechanisms.

Regarding composition, the lack of significant differences in protein or fat between treatments mirrors the results of Liu et al. (2020), who found that *T. molitor* larvae maintained a stable nutrient composition even when their diets were supplemented with fruits and vegetables [20]. This stability likely reflects the species’ capacity for nutrient regulation and homeostasis, as mealworms can adjust feeding behavior and digestive efficiency to maintain balanced nutrient intake despite variation in diet composition [18]. Given that apple pomace was provided as a supplemental hydration source rather than a full feed replacement, this may also explain the absence of large compositional shifts between treatments [20]. However, the lower moisture content observed in AP-fed larvae suggests that the type and accessibility of water source, i.e., direct hydration versus fiber-bound moisture, can influence larval hydration dynamics. The high fiber and polyphenol content of apple pomace may have further limited water extraction efficiency, resulting in lower internal moisture levels despite its overall high moisture percentage [9].

While this study found no compositional enhancement from apple pomace supplementation, other agricultural byproducts such as olive pomace, cabbage leaves, and mushroom substrate have been shown to increase larval protein content [5,12,19]. Li et al. (2013) reported an 8% increase in crude protein when cabbage leaves were incorporated into a wheat bran diet, while Ruschioni et al. (2020) found nearly a 10% improvement with olive pomace supplementation [5,19]. Similarly, Zhang et al. (2019) demonstrated that agricultural byproducts such as soybean meal, distiller’s grains, and spent mushroom substrate enhanced larval protein content compared to controls [12]. Among these, corn stover yielded the highest protein concentration and proved the most cost-effective option, underscoring the potential economic and sustainability benefits of integrating agricultural residues into insect rearing systems. This highlights the variability in nutritional impact among byproducts, emphasizing the need for broader evaluation of agro-industrial residues as feed substrates.

## 5. Conclusions

In summary, supplementing mealworm diets with apple pomace did not alter growth rate or proximate composition but improved larval survival by almost 10% compared with the control (73% vs. 67%). This enhancement may reflect antioxidant protection during metamorphosis, though further biochemical validation is warranted. Given its abundance, low cost, and nutritional profile, apple pomace could be feasibly integrated into large-scale mealworm production without negatively affecting performance. Incorporating such byproducts supports sustainable, circular food systems by reducing waste while contributing to scalable protein production.

## Figures and Tables

**Table 1:** Growth rate of mealworm larvae over a 4-week period where a standard wheat-based worm chow was supplemented with apple pomace in place of standard water beads.

	Water Bead (WB)		Apple Pomace (AP)	
Interval Week	Larval wt. (mg)	Δ wt. (mg)	Larval wt. (mg)	Δ wt. (mg)
Initial	5.66 ± 0.32 ^d^		5.50 ± 0.32 ^d^	
1	8.37 ± 0.32 ^c^	2.71	8.06 ± 0.32 ^c^	2.65
2	8.97 ± 0.32 ^b^	0.60	8.89 ± 0.32 ^b^	0.83
3	9.55 ± 0.32 ^b^	0.56	9.17 ± 0.32 ^b^	0.27
4	10.5 ± 0.32 ^a^	0.90	10.0 ± 0.32 ^a^	0.82
% Weight gain	84.99 ± 0.21		81.86 ± 0.24	

Note: Values are the LS Means (mg ± SE) of 10 randomly selected mealworm larvae and different superscripts within a specific parameter indicate significant differences (*p* < 0.05). There were no significant differences in weight gain over time between apple pomace and water bead (control) treatments (ANOVA, *p* > 0.05).

**Table 2:** Survival and mortality ^a^ of mealworm larvae fed a wheat-based worm diet supplemented with apple pomace (AP) in place of standard water beads (WB) over a 4-week period (N = 4774).

	Pupation/Mortality/Survival Counts				Life Table Method (Actuarial)		Kaplan–Meier	
Treatment	Interval (Weeks) ^b^	Pupation Count	Mortality Count	Live Larvae	Survival %	Mortality %	Survival %	Mortality %
AP	0	0	0	2386	100.00%	0.00%	100.00%	0.00%
	1	16	82	2288	96.55%	3.45%	96.56%	3.44%
	2	318	129	1841	90.70%	9.30%	91.12%	8.88%
	3	555	70	1216	86.64%	13.36%	87.65%	12.35%
	4	668	136	412	73.28%	26.72%	77.85%	22.15%
WB	0	0	0	2388	100.00%	0.00%	100.00%	0.00%
	1	24	84	2280	96.46%	3.54%	96.48%	3.52%
	2	352	144	1784	89.86%	10.14%	90.39%	9.61%
	3	548	115	1121	83.02%	16.98%	84.56%	15.44%
	4	583	163	375	66.71%	33.29%	72.27%	27.73%
Log-Rank Test of Equality over Treatment ^c^						Chi-Square = 12.80, *p* = 0.0003		

^a^ Values represent aggregated weekly cohort totals rather than replicate-level data; therefore, measures of dispersion (e.g., SD or SEM) are not shown. Variation among replicates (n = 6 per treatment) was incorporated into the repeated-measures ANOVA, supplemented using Life Table and Kaplan–Meier survival analyses. ^b^ Week 0 marks the conclusion of the acclimation period and serves as the baseline for all measurements. ^c^ Both categorical survival analyses were followed by a Log-Rank test for Equality of Strata, with treatment cohorts defined as the strata. A significant *p*-value (*p* < 0.05) indicates a statistically different survival outcome between cohorts.

**Table 3:** Proximate composition, dry basis (d.b.), of commercial wheat-based worm feed, apple pomace, and mealworm larvae after a 4-week feeding study with a standard wheat-based diet with water beads (WB) or apple pomace (AP).

Material	Moisture (g/100 g)	Protein (g/100 g d.b.)	Fat (g/100 g d.b.)	Ash (g/100 g d.b.)
Wormy Worm Chow	10.95 ± 0.25	8.46 ± 0.12	6.13 ± 0.09	1.71 ± 0.21
Apple Pomace	85.86 ± 0.89	1.20 ± 0.10	3.19 ± 2.22	0.04 ± 0.04
Mealworm (AP)	63.89 ± 0.01 ^a^	33.77 ± 0.06	52.27 ± 0.09	0.16 ± 0.00
Mealworm (WB)	66.89 ± 0.01 ^b^	34.39 ± 0.03	51.94 ± 0.15	0.20 ± 0.00

Data are given as means ± SD. Mean values with different letters indicate significant differences (*p*< 0.05).

## Data Availability

The original contributions presented in this study are included in the article. Further inquiries can be directed to the corresponding authors.

## Abbreviations

The following abbreviations are used in this manuscript:

AP	Apple pomace
D.B.	Dry basis
ECI	Efficiency of conversion of ingested feed
WB	Water bead
